# Chinese Medicine, Succinum, Ameliorates Cognitive Impairment of Carotid Artery Ligation Rats and Inhibits Apoptosis of HT22 Hippocampal Cells *via* Regulation of the GSK3β/β-Catenin Pathway

**DOI:** 10.3389/fphar.2022.867477

**Published:** 2022-06-15

**Authors:** Chongqi Wei, Ziqiang Zhu, Jia-ni Zheng, Yunqing Lu, Cheng Cao, Suchen Qu, Mengqiu Liu, Xue-er Meng, Qianyin Lou, Qingqing Wang, Jin-ao Duan, Er-xin Shang, Zhenxiang Han, Yue Zhu

**Affiliations:** ^1^ Jiangsu Key Laboratory for High Technology Research of TCM Formulae and Jiangsu Collaborative Innovation Center of Chinese Medicinal Resources Industrialization, Nanjing University of Chinese Medicine, Nan Jing, China; ^2^ Department of Neurology and Rehabilitation, Seventh People’s Hospital of Shanghai University of Traditional Chinese Medicine, Shanghai, China

**Keywords:** succinum, vascular dementia, apoptosis, Wnt/GSK3β, mineral medicine

## Abstract

Succinum is an organic mineral formed from the resin of ancient coniferous and leguminous plants, which is applied for tranquilizing mood, promoting blood circulation, and removing blood stasis in Chinese medicine. For quite a long time, the modern research of succinum mainly focuses on the study of physical and chemical properties and authenticity identification while few reports on its medicinal mechanism. In current study, we evaluated different solvent extracts of succinum on carotid artery ligation rats mimicking vascular dementia. It was found that ethyl acetate extracts of succinum significantly improved the learning and memory abilities of model rats and inhibited neuronal apoptosis in the hippocampus. On a mice hippocampal neuronal cell line (HT22), ethyl acetate extracts of succinum also exerted better action trend in inhibiting cell apoptosis induced by oxygen glucose deprivation (OGD). By using XAV-939 on both *in vivo* and *in vitro* studies, it was found that ethyl acetate extracts of succinum might exert these functions by regulating the GSK3β/β-catenin pathway. These studies revealed the neuronal function of succinum, which explained the traditional effects of succinum and provided more modern scientific basis for its clinical application.

## Introduction

Vascular dementia (VD) is a cognitive disorder based on cerebral nerve damage caused by cerebrovascular diseases. Patients often have clinical manifestations such as impairment of memory, cognition, and executive function after cerebrovascular diseases. The neuropathological classification of VD includes dementia caused by ischemic and hemorrhagic brain damage and hypoxia–hypoperfusion dementia. Now, VD has become the second highest incidence of dementia after Alzheimer’s disease. Prevention and treatment of cerebrovascular disease and related risk factors are regarded as the fundamental method of VD treatment, which includes antiplatelet aggregation, lipid reduction, prevention and treatment of hypertension and diabetes, and so on. In addition, donepezil, a cholinesterase inhibitor, and memantine, a non-competitive NMDA receptor antagonist, may improve cognitive function in VD patients. In addition, vitamin E, vitamin C, Ginkgo biloba extracts, piracetam, and nicergoline might have some adjuvant therapeutic effects. No specific drug has approved to treat VD (Smith, 2017).

It is now recognized that cerebrovascular dysfunction is an important pathological factor of VD, and ischemia and hypoxia caused by cerebrovascular dysfunction lead to cognitive decline. The hippocampus is closely associated with learning and memory, which is particularly vulnerable to ischemic damage (Zhu et al., 2018). Ischemia and hypoxia of the brain lead to the accumulation of free radicals and excitatory amino acids in blood supply area of middle cerebral artery such as the cortex, hippocampus, and striatum, which results in intracellular calcium overload and neuronal apoptosis, even necrosis (van der Flier et al., 2018; Wang et al., 2020; Kuang et al., 2021). Since the GSK3β/β-catenin signaling pathway plays a critical role in neuronal cell growth, it is regarded as an important signaling pathway in the pathological mechanism of VD (Foulquier et al., 2018).

Fossil Chinese medicinal materials are a unique type of Chinese medicinal materials for treating neurological disease including dementia. They are derived from ancient plants and animals buried underground and transformed with the movement of the Earth’s crust for hundreds of millions of years. Succinum is one of the fossil Chinese medicinal materials widely used in clinics, which is a resin from Mesozoic Cretaceous to Cenozoic Tertiary pine and cypress, which is an organic mixture formed by geological action. The earliest medicinal records were collected by Ming Yi Bie Lu in 450 AD (Tao, 1986), and the function has been summarized as tranquilizing the mind, promoting blood circulation to remove blood stasis, and diuresis to relieve stranguria by Zhong Hua Ben Cao (Hu et al., 1998). In the masterpieces of internal medicine of Chinese Medicine ZaBing YuanLiu XiZhu written by Shen Jin-ao in the Qing dynasty, succinum was used for treating “phlegm obsessing with the heart, speaking like an idiot but forgetting anything,” which is similar to Alzheimer’s disease (Shen, 1994). Different from other Chinese medicinal materials applied for AD such as ginseng and Poria, succinum is especially suitable for the treatment of vascular dementia due to its character of promoting blood circulation and removing blood stasis. However, the modern research on succinum mainly focuses on the identification of authenticity while few reports on elucidation of the pharmacological action mechanism.

Based on medical records of succinum in Chinese medicine, we developed a cerebral ischemia VD animal model by ligating carotid artery of rats and evaluated the effect of succinum on improving learning and memory of rats. Furthermore, a mouse hippocampal neuronal HT22 cell model under glucose and oxygen deprivation was developed to evaluate the antiapoptotic effect of succinum. Based on the results of *in vivo* and *in vitro* studies, we found that ethyl acetate extracts of succinum significantly improved the learning and memory abilities of model rats and inhibited neuronal cell apoptosis of HT22 under OGD condition *via* the regulation of the GSK3β/β-catenin pathway. Based on these findings, succinum might be used as a supplement therapy for patients with cerebral ischemia VD, providing a scientific basis for drug development of succinum.

## Materials and Methods

### Preparation of Extracts

Succinum samples were purchased from Buchang Pharmaceutical Co., Ltd. The morphology of succinum and the voucher number can be found in Supplementary Figure S1. Extracts with different polarities of succinum were prepared by the following procedures. Pulverized succinum (50 g) was mixed with 500 ml petroleum ether and refluxed for 8 h. The supernatant was removed, and the residue was added fresh to 500 ml petroleum ether for a second reflux. The supernatants from two refluxes were combined, and the solvent was removed by rotary evaporation. The residues were extracted with ethyl acetate, n-butanol, and 70% ethanol sequentially. The extracts were characterized using UPLC-QTOF-MS/MS, and the representative chromatogram of each type extract is displayed in Supplementary Figure S2. The content of representative compound of isopimarane diterpenoid in succinum is about 0.88 μg/g. This information set up the quality control standard of succinum extracts for further biological evaluation.

### Animals and Housing Conditions

Male SD rats (7–8 weeks old, 280–300 g) were purchased from Shanghai SLAC Laboratory Animal Co., Ltd. SD rats were raised in the SPF environment of the 12 h light/dark cycle, at 22–25°C, and a humidity of 40–70% in the animal center of Nanjing University of Chinese Medicine. All procedures for treating animals were following the guide for the care and use of laboratory animals approved by the institutional animal care and use committee. The experimental procedures also conformed to the guidelines of the “Principles of Laboratory Animal Care” (NIH publication No. 80-23, revised 1996). In particular, all procedures were performed to minimize the number of experimental animals and possible injuries.

### Cerebral Ischemic Rat Model Development

The rats were habituated to the housing conditions 7 days before model development. According to the literature (Shi et al., 2018), the cerebral ischemic rat model was established by occluding unilateral common carotid artery. In short, animals were anesthetized by 10% chloral hydrate intraperitoneal injection (300 mg/kg) and fixed on an operating table. The skin of the neck was disinfected, and the incision was made on the midline of the neck to expose the arteries. The muscles were separated from connective tissue. The right artery was ligated at the proximal and distal end with an operation line. The vagus was protected from damage. The rats in the sham group received the same operation without unilateral common carotid artery ligation. During the operation, the animals were remained warm to maintain the body temperature at 37 ± 0.5°C. After 2 weeks of recovery, the Morris water maze test was used to determine whether the model was successfully established, and the rats were treated with different drugs for another 2 weeks.

### Experimental Animal Grouping and Drug Treatment

The animals were randomly divided into 12 groups, with eight rats in each group. The control group animals were given saline intragastrically. The positive control group was given huperzine A (0.05 mg/kg/day) (Damar et al., 2016). The eight treatment groups were given petroleum ether, ethyl acetate, n-butanol, and 70% ethanol extracts of succinum, respectively, at dosages of 0.21 g/kg/day and 0.42 g/kg/day, which is equivalent to the human dosage of 2 and 4 g of crude succinun. Animals were treated with different drugs for 14 days.

To explore the role of the GSK3β/β-catenin pathway in ameliorating memory impairment of carotid artery ligation rats, model rats were treated with XAV-939 (β-catenin inhibitor, 40 mg/kg, intraperitoneal injection) in addition to the treatment of ethyl acetate fraction of succinum (0.42 g/kg/day) (Jean LeBlanc et al., 2019; Cheng et al., 2020).

### Morris Water Maze Test

The learning and memory behaviors were evaluated by using the Morris water maze test, and the details could be referred to our previous publication (Zhu Y. et al., 2019). In detail, the Morris water maze device was filled with water and divided into four equal quadrants. The temperature of water was adjusted to 25°C. Ink was put into water so that the animal could not see the platform. A round platform was placed in the fourth quadrant and immersed 1 cm below the water surface.

In the training session, the rats entered the water from the second quadrant facing the wall of the water tank and were given a free swimming for 120 s. If rats could not find the platform during the training time in 120 s, the rats would be guided to the platform and stayed for 30 s. The training session lasted 4 days consecutively, and the rats were placed at different starting positions of quadrant in each day. The test session was carried out on the fifth and sixth day. In the first test, the rats were put into the water facing the cylinder wall from the third quadrant, and the rats were given 60 s to find the platform. During this session, the time and routine were recorded. After 24 h, the platform was removed and the rats were put into the water facing the cylinder wall from the fourth quadrant and swam freely for 60 s. The time to swim in the target quadrant where the platform was previously placed, and the number of times crossing over the platform site was recorded. All the data were analyzed by using ANY-maze software.

### TUNEL Assay

After behavioral tests, the rats were anesthetized and decapitated, and the brains were removed and fixed in 4% paraformaldehyde for observation of tissue apoptosis in the hippocampus by TUNEL assay, according to the manufacturer’s instruction of the TdT-FragELTM DNA Fragmentation Detection Kit (TUNEL, Vazyme Biotech Co.,Ltd, China). The brain tissue sections were dewaxed with xylene and dehydrated with gradient ethanol. Then, 20 μg/ml protease K without DNase was added to the sections and incubated at 25^o^C for 20 min. The sections were treated with 50 μL TdT buffer, incubated at room temperature for 60 min, and sealed with a fluorescent mounting tablet (Dako, Denmark). Hippocampal dentate gyrus in each section was detected for analysis under the microscope at ×40 magnification for taking pictures. The apoptosis of HT22 cell cultures were also determined according to the same manufacture instruction of TUNEL assay kit.

### Immunohistochemistry

After behavioral tests, the rats were anesthetized and decapitated, and the brains were removed and fixed in 4% paraformaldehyde and followed by dehydration, paraffin embedding, and brain slice preparation. Brain slices were dewaxed with xylene, dehydrated in an ethanol gradient, and then subjected to antigen repair in sodium citrate buffer solution for 20 min. This was followed by dehydrogenation with 3% hydrogen peroxide. Brain slices were blocked with 5% bovine serum albumin (BSA) in 0.2% PBS-T for 2 h at room temperature and incubated with rabbit anti-MAP2 (AF10717, 1:100, AiFang biological, China) at 4°C for 36 h, then removed from the refrigerator and incubated at room temperature for 1 h. After washing three times, brain slices were incubated with Alexa Fluor^®^ 488 antirabbit antibody (1:1,000, Invitrogen) for 1 h. DAPI (1:10,000) staining was performed for 5 min and sealed with a fluorescent mounting tablet (Dako, Denmark). Hippocampal dentate gyrus in each section was examined under a microscope at ×20 and ×40 magnifications for analysis.

### Cell Cultures

The mice hippocampal neuronal cell line (HT22) was purchased from Cyagen Biotechnology Co.,Ltd (Guangzhou, China). The cells were cultured in Dulbecco’s modified Eagle’s medium (Corning Industrial Technology Co.,Ltd., United States) supplemented with 10% FBS, 100 U/mL penicillin, and 100 μg/ml streptomycin. The cell culture conditions were maintained in the incubator at 37 C with 95% air and 5% CO_2_. The culture medium was changed every 48 h, and the cells were sub-cultured when the cell confluence reached 70%. All reagents used for cell culture were purchased from Thermo Fisher Scientific (Invitrogen, Carlsbad, CA).

### Oxygen–Glucose Deprivation Cell Model Development

The HT22 cell model of glucose and oxygen deprivation was developed according to publication (Vahabzadeh et al., 2015). In detail, the medium of HT22 cell culture was replaced with glucose-free DMEM (Sigma-Aldrich, United States), and the cells were cultured in a small anaerobic chamber filled with 95% (v/w) N2 and 5% (v/w) CO_2_. The control group cells were cultured under normal conditions as mentioned before.

### MTT Assay

The HT22 cells were seeded in a 96-well plate at a density of 20 cell/μL. MTT with a final concentration of 5 μg/ml was added to each well and incubated at 37°C for 3 h. Then, MTT was sucked out and 150 μL of DMSO was added to a 96-well plate and incubated for 30 min on the shaker at 37°C. The absorbance at 570 nm was measured using a microplate spectrophotometer.

### Western Blotting Analysis

The experiment of sodium dodecyl sulfate polyacrylamide gel electrophoresis (SDS-PAGE) and Western blotting analysis was carried out to protocol, which can be found in our previous publication (Zhu et al., 2017). The total protein samples extracted and lysed from animal tissues or cell cultures by RIPA (RIPA, Beyotime Biotech Co.,Ltd, China) were separated by 10% polyacrylamide gels and transferred to the nitrocellulose filter membrane. The primary antibodies used were rabbit monoclonal anti-MAP2 (4,542, 1:2,000; Cell Signaling Technology), rabbit monoclonal anti-GSK3β (9,315, 1:2,000; Cell Signaling Technology), rabbit monoclonal anti-p-GSK-3β (9,323, 1:2,000; Cell Signaling Technology), rabbit monoclonal anti-β-catenin (8,480, 1:2,000; Cell Signaling Technology), rabbit monoclonal anti-p-β-catenin (9,561, 1:2,000; Cell Signaling Technology), mouse monoclonal anti-caspase-3 (66470-2-1g, 1:2,000; Proteintech), rabbit monoclonal anti-cleaved-caspase-3 (9,664, 1:2,000; Cell Signaling Technology), mouse monoclonal anti-Bax (sc-7,480, 1:1,000; Santa Cruz Biotechnology), mouse monoclonal anti-Bcl-2 (sc-7,382, 1:1,000; Santa Cruz Biotechnology), and rabbit polyclonal anti-β-Actin (beta-actin) (bs-0061R, 1:10,000; Bioss). The secondary antibody used was antirabbit IgG antibody conjugated with horseradish peroxidase (1:10,000, Boster) and antimouse IgG antibody conjugated with horseradish peroxidase (1:10,000, Boster). The band was displayed by an enhanced chemiluminescence (ECL) Western blotting substrate kit (Tiannen, China), and the density was determined by using the Bio-Rad Imaging System (ChemiDoc™XRS+, Bio-Rad, Hercules, CA).

### Data Analysis

Statistical analysis was performed using one-way or two way ANOVA analysis (version 13.0, SPSS, IBM Corp., Armonk, NY) followed by a Bonferroni *post hoc* analysis if appropriate. Before ANOVA analysis, a normal distribution test was carried out. The results were expressed as mean ± standard error of the mean, where *n* = 3–8. Statistically significant changes were represented by a *p*-value. In detail, *p*-value < 0.05 was considered statistically significant, and *p* < 0.01 was considered highly statistically significant.

## Results

### Extracts of Succinum Ameliorated Learning and Memory Impairment on Carotid Artery Ligation Rats

**FIGURE 1:**
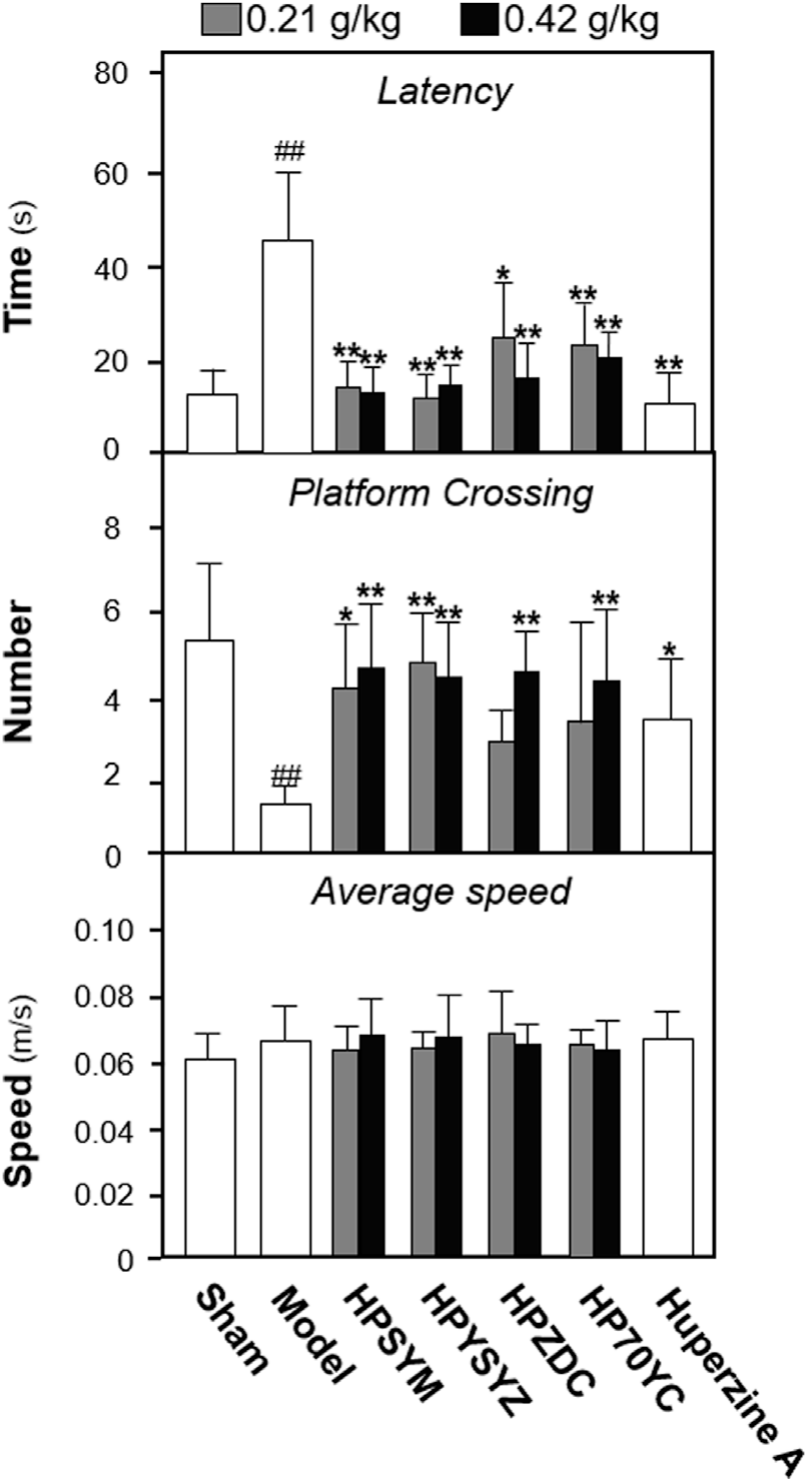
Extracts of succinum ameliorated learning and memory impairment on carotid artery ligation rats. The effect of succinum extracts on the latency time, platform crossing number, and average speed of carotid artery ligation rats. Values are expressed as mean ± SEM (*n* = 8). Comparisons between groups were carried out by a one-way ANOVA followed by a *post hoc* Bonferroni test. ^##^ *p* < 0.01 (compared with the sham control group), *p < 0.05, and **p < 0.01 (compared with the carotid artery ligation model group).

To explore the active ingredients of succinum on ameliorating learning and memory impairment of carotid artery ligation rats, succinum powders were sequentially extracted by solvent of petroleum ether, ethyl acetate, n-butanol, and 70% ethanol, and the extracts were intragastrically administered for model animals for 14 days. The Morris water maze test is applied for evaluating learning and memory of rats, which is separated into the place navigation test and the spatial probe test. As displayed in Figure 1, the time of latency in the model group was significantly higher, while the number of platform crossing was lower both compared with the control sham group (*p* < 0.01), which suggested that carotid artery ligation induced learning and memory impairment. Compared with the VD model group, different solvent extracts of succinum all decreased the latency time and increased the platform crossing number of the model animals (*p* < 0.01). Among them, ethyl acetate extracts of succimum exerted the strongest effect in ameliorating learning and memory impairment of model animals. In addition, the swimming speed of the animals was determined, and there was no significant difference found between the different group animals, which indicated that succinum extracts exerted no obvious effect on locomotor ability of animals.

### Ethyl Acetate Extracts of Succinum-Reduced Hippocampal Apoptosis and Neuronal Loss of Carotid Artery Ligation Rats

**FIGURE 2:**
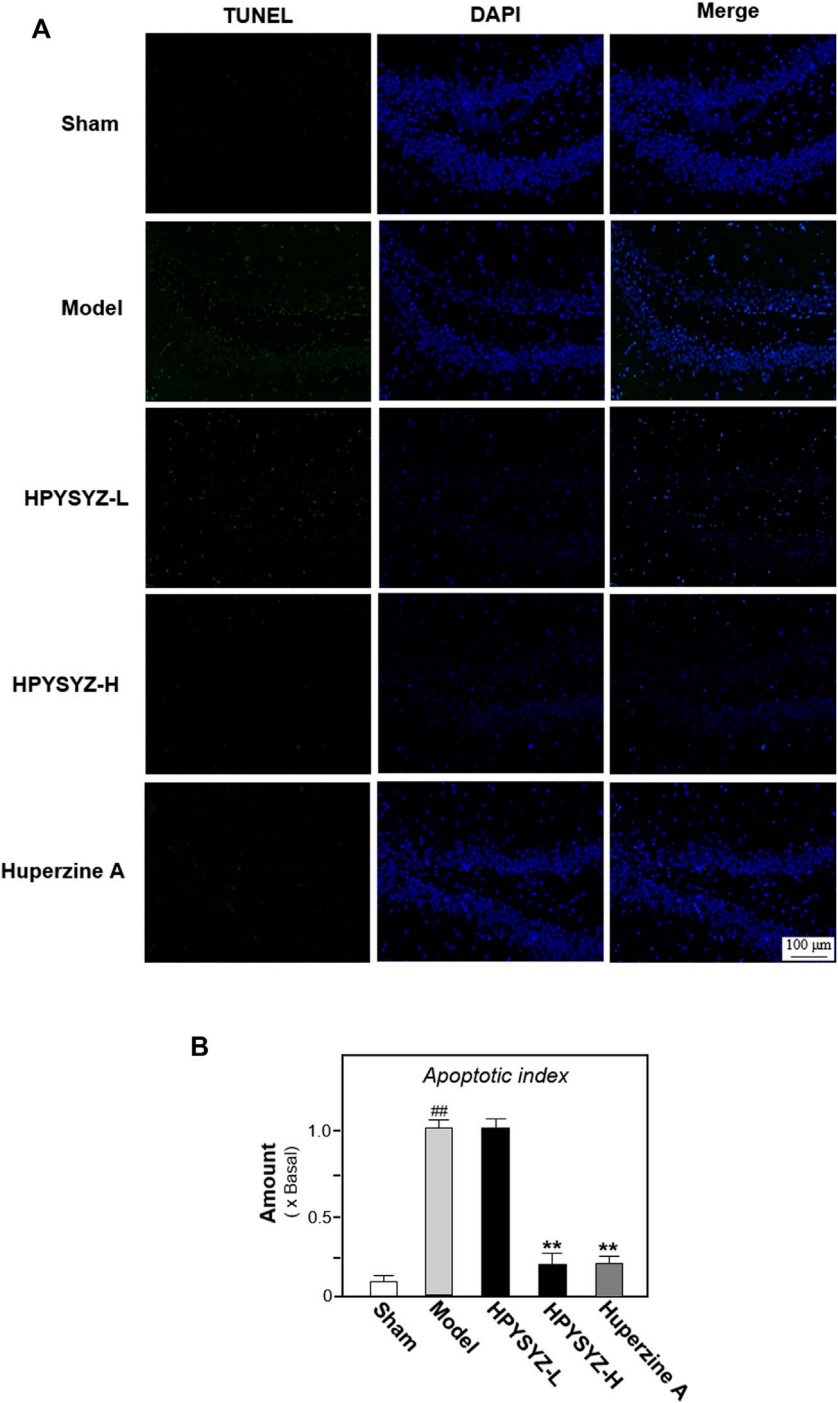
Ethyl acetate extracts of succinum reduced the apoptosis in the hippocampus of carotid artery ligation rats. **(A)** Apoptosis of the hippocampal dentate gyrus of rats in the sham control group, model group, low dose and high dose treatment of ethyl acetate extracts of succinum groups, and positive control huperzine A group were determined by TUNEL assays. **(B)** Percentage of TUNEL staining positive cells to DAPI-staining positive cells was calculated for apoptosis evaluation. The low dose treatment of ethyl acetate extracts of succinum (HPYSYZ-L) was set as 0.21 g/kg/d, and the high dose treatment (HPYSYZ-H) was set as 0.42 g/kg/d (*n* = 3). The analysis was performed from two brain sections of one rat and three rats from one group (mean ± SD, *p < 0.05, and **p < 0.01).

**FIGURE 3:**
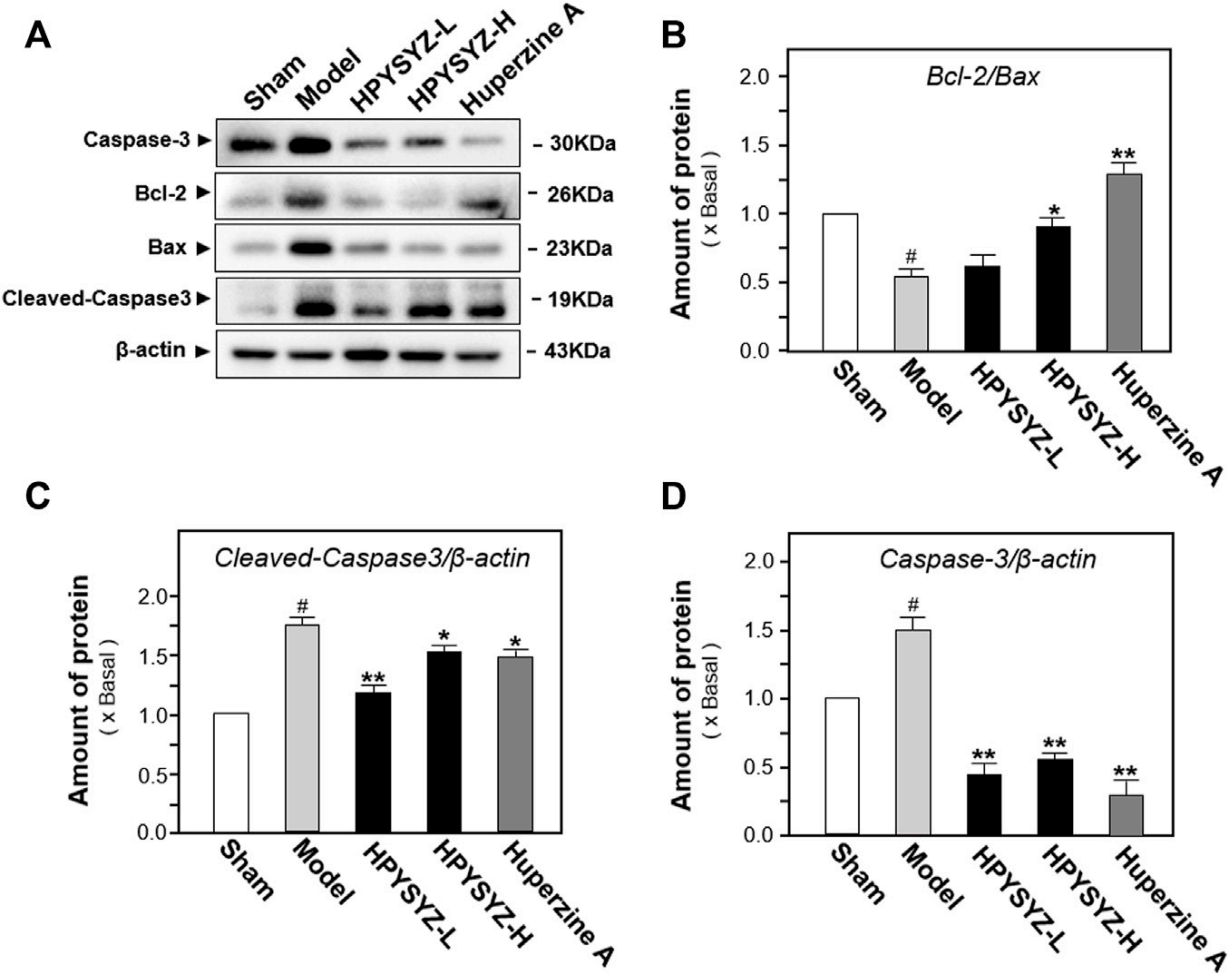
Ethyl acetate extracts of succinum regulated the caspase-dependent pathway in the hippocampus of carotid artery ligation rats. **(A)** Effects of ethyl acetate extracts of succinum at low dosage (HPYSYZ-L) and high dosage (HPYSYZ-H) on the expressions of caspase-3, cleaved-caspase-3, and Bax and Bcl-2 of hippocampus of carotid artery ligation rats were evaluated by Western blotting analysis. The representative images are displayed (*n* = 5). **(B)** Expression of caspase-3 was analyzed and the expression of β-actin was applied for normalization. Expression of cleave-caspase-3 was analyzed and the expression of β-actin was applied for normalization. **(D)** Expressions of Bax and Bcl-2 were analyzed and the expression of β-actin was also used for normalization. The expression ratio of Bcl-2 to Bax was calculated. Values are expressed as the percentage of the control group (no drug treatment normal mice) as mean ± SEM (n = 5). Comparisons between groups were analyzed by a one-way ANOVA followed by a *post hoc* Bonferroni test. ^##^ *p* < 0.01 (compared with control group), *p < 0.05, and **p < 0.05 (compared with the model group).

Based on the results of behavioral tests, the hippocampal tissues of the ethyl acetate extract of succimum-treated rats were selected and sacrificed, and the entire brain was isolated, and the apoptosis condition was determined by using TUNEL assays. Figure 2 displays carotid artery ligation significantly induced apoptosis in the hippocampal dentate gyrus of model rats with the high level of green fluorescence compared with the sham control group. Treatment of ethyl acetate extracts of succinum significantly decreased the level of green fluorescence in the hippocampal dentate gyrus compared with the untreated model rats. In addition, the effect of ethyl acetate extracts of succinum was evaluated by the expression of apoptosis-related proteins including caspase-3, cleaved-caspase-3, and Bcl-2 and Bax. As shown in Figure 3, the expressions of caspase-3 and cleaved-caspase-3 protein were increased, while the ratio of Bcl-2/Bax was decreased in the hippocampus of carotid artery ligation model rats compared with the sham control group rats (*p* < 0.05). Ethyl acetate extracts of succinum downregulated the expressions of caspase-3 and cleaved-caspase-3 while upregulated the ratio of Bcl-2/Bax in the hippocampus of model rats (*p* < 0.05). These data suggested that ethyl acetate extracts of succinum prevented neuronal cells of the hippocampus in carotid artery ligation model rats from apoptosis.

**FIGURE 4:**
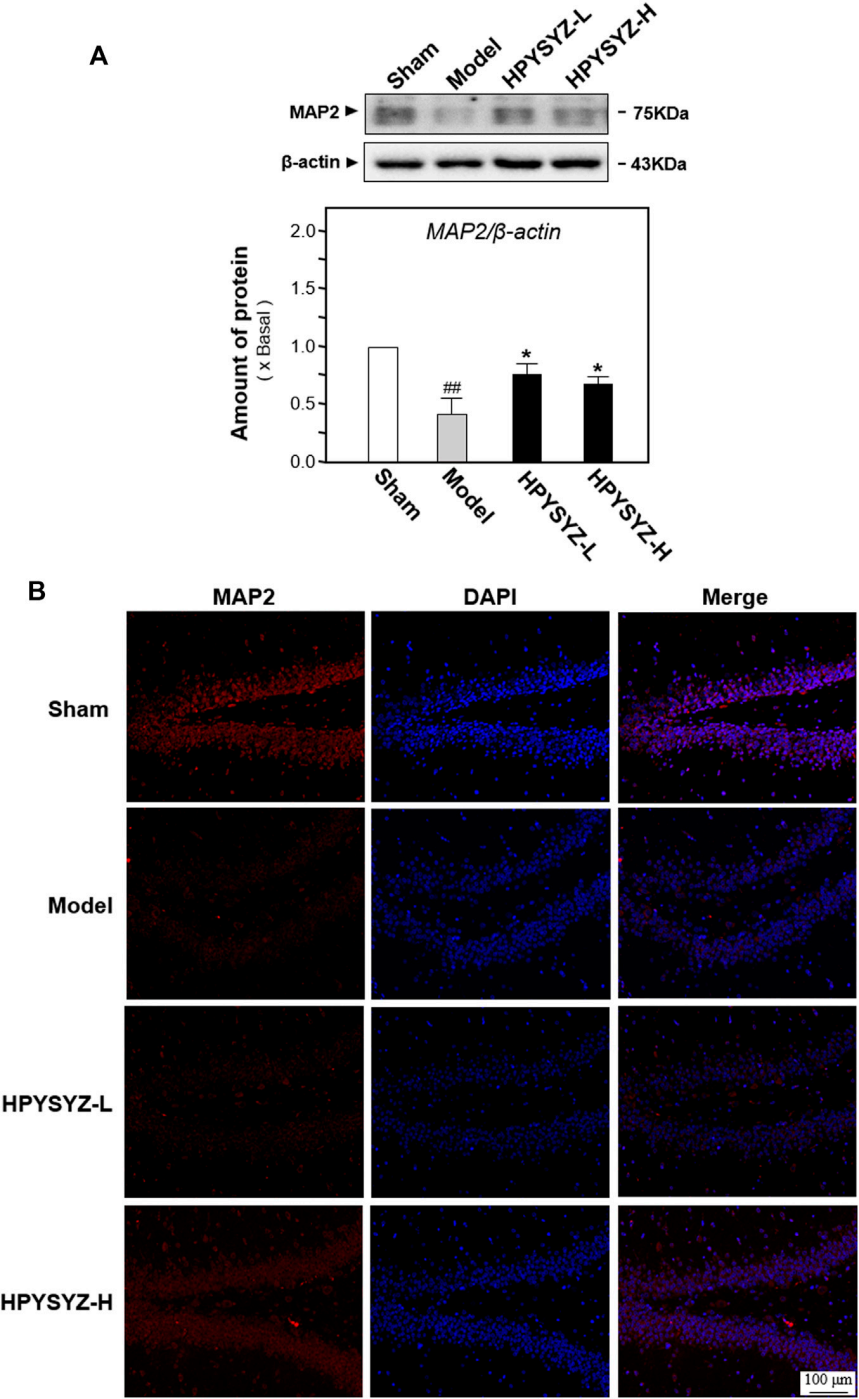
Ethyl acetate extracts of succinum regulated the expression of neuronal marker MAP2 in the hippocampus on carotid artery ligation rats. **(A)** Expressions of neuronal cell marker MAP2 were determined in the hippocampus of carotid artery ligation rats by Western blotting analysis. Values are expressed as mean ± SEM (*n* = 5). Comparisons between groups were carried out by a one-way ANOVA followed by a *post hoc* Bonferroni test. ^##^ *p* < 0.01 (compared with the normal control group), ^*^ *p* < 0.05, and ^**^ *p* < 0.01 (compared with the carotid artery ligation model group). **(B)** Expressions of neuronal cell marker MAP2 were determined in the hippocampus of carotid artery ligation rats by immunohistochemistry analysis. The low dose treatment of ethyl acetate extracts of succinum (HPYSYZ-L) was set as 0.21 g/kg/d, and the high dose treatment (HPYSYZ-H) was set as 0.42 g/kg/d (*n* = 3).

In addition to apoptosis evaluation, the effect of ethyl acetate extracts of succinum on neuronal cell loss was also evaluated by determining the expressions of microtubule-associated protein 2 (MAP2), the biomarker of neuronal cells, in the hippocampus of animals. Figures 4A,B display carotid artery ligation decreased the expressions of MAP2 of model rats compared with the sham group rats. Comparatively, treatment of ethyl acetate extracts of succinum significantly upregulated the expressions of MAP2 of model animals compared with the untreated model group rats. These data indicated that ethyl acetate extracts of succinum could attenuate the neuronal loss of the hippocampus of carotid artery ligation rats.

### Ethyl Acetate Extracts of the Succinum-Regulated GSK3β/β-Catenin Pathway in the Hippocampus of Carotid Artery Ligation Rats

**FIGURE 5:**
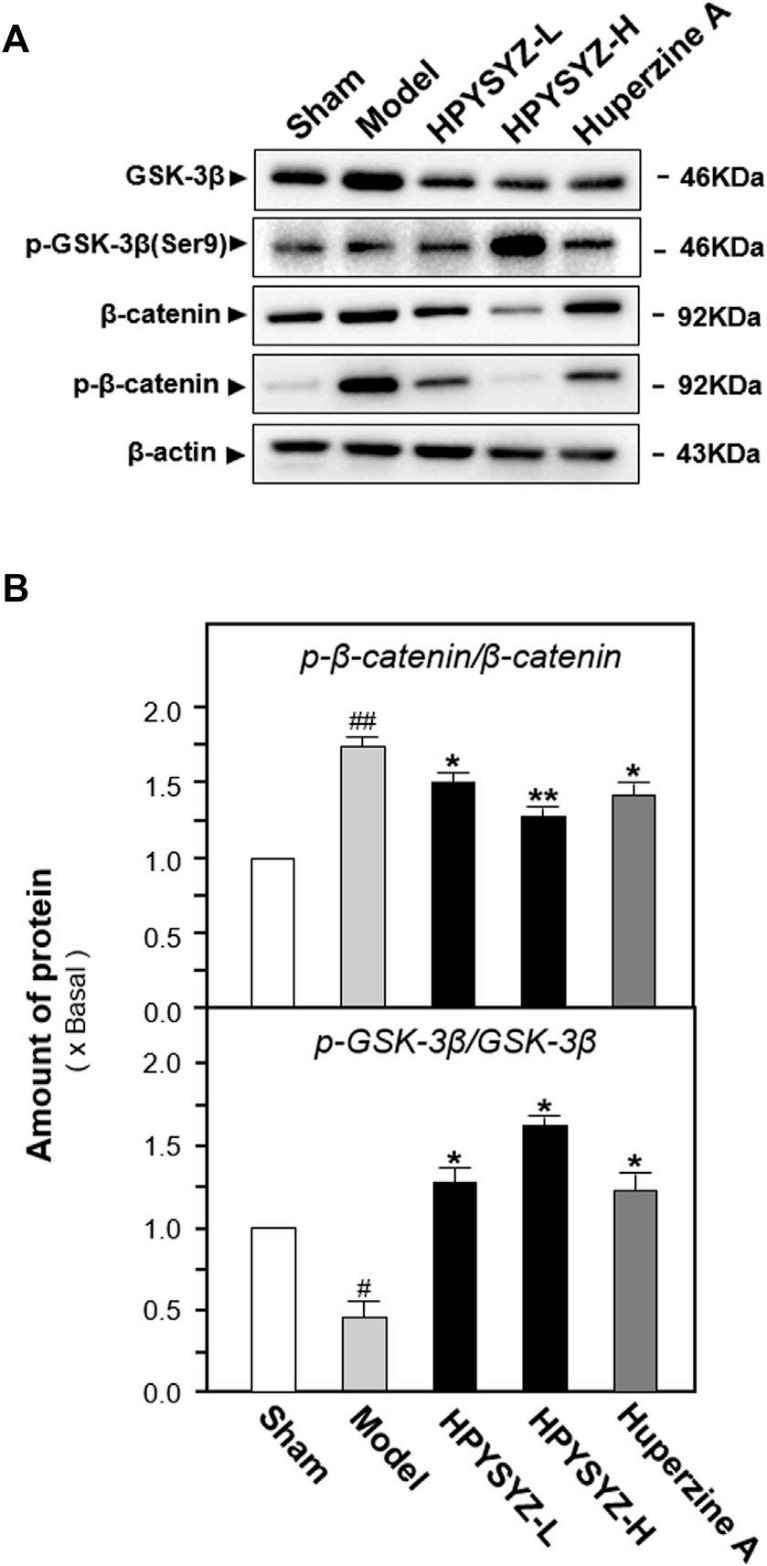
Ethyl acetate extracts of succinum regulated the GSK3β/β-catenin signaling pathway in the hippocampus of carotid artery ligation rats. **(A)** Effects of ethyl acetate extracts of succinum at low dosage (HPYSYZ-L) and high dosage (HPYSYZ-H) on the expressions of GSK3β, p-GSK3β (Ser9), β-catenin, p-β-catenin, and β-actin of hippocampus of carotid artery ligation rats were evaluated by Western blotting analysis. The representative images are displayed (*n* = 5). **(B)** Expression ratio of p-β-catenin to β-catenin was calculated, and the expression of β-actin was applied for normalization. Expression ratio of p-GSK3β (Ser9) to GSK3β was calculated, and the expression of β-actin was applied for normalization. Values are expressed as the percentage of the control group (no drug treatment normal mice) as mean ± SEM (*n* = 5). Comparisons between groups were analyzed by a one-way ANOVA followed by a *post hoc* Bonferroni test. ^##^ *p* < 0.01 (compared with the control group), *p < 0.05, and **p < 0.05 (compared with the model group).

To explore the possible signaling pathway of ethyl acetate extracts of succinum on regulation of hippocampal apoptosis, the expressions of GSK3β/β-catenin pathway-related kinases including GSK3β, p-GSK3β, β-catenin, and p-β-catenin were determined in the hippocampus of carotid artery ligation rats. As shown in Figures 5A,B, the ratio of p-GSK3β to GSK3β was decreased in the hippocampus of carotid artery ligation rats compared with the sham group, while the ratio of p-β-catenin to β-catenin was increased in the hippocampus compared with the sham group (*p* < 0.01). Compared with model group rats, treatment of ethyl acetate extracts of succinum at two dosages both upregulated the expression ratio of p-GSK3β to GSK3β while downregulated the ratio of p-β-catenin to β-catenin compared with model group rat. (*p* < 0.05). These data suggested that ethyl acetate extracts of succinum activated GSK3β/β-catenin pathway protein in the hippocampus of carotid artery ligation rats.

### Ethyl Acetate Extracts of Succinum Ameliorated Learning and Memory Impairment and Hippocampus Apoptosis of Carotid Artery Ligation Rats by Regulating the GSK3β/β-Catenin Signaling Pathway

**FIGURE 6:**
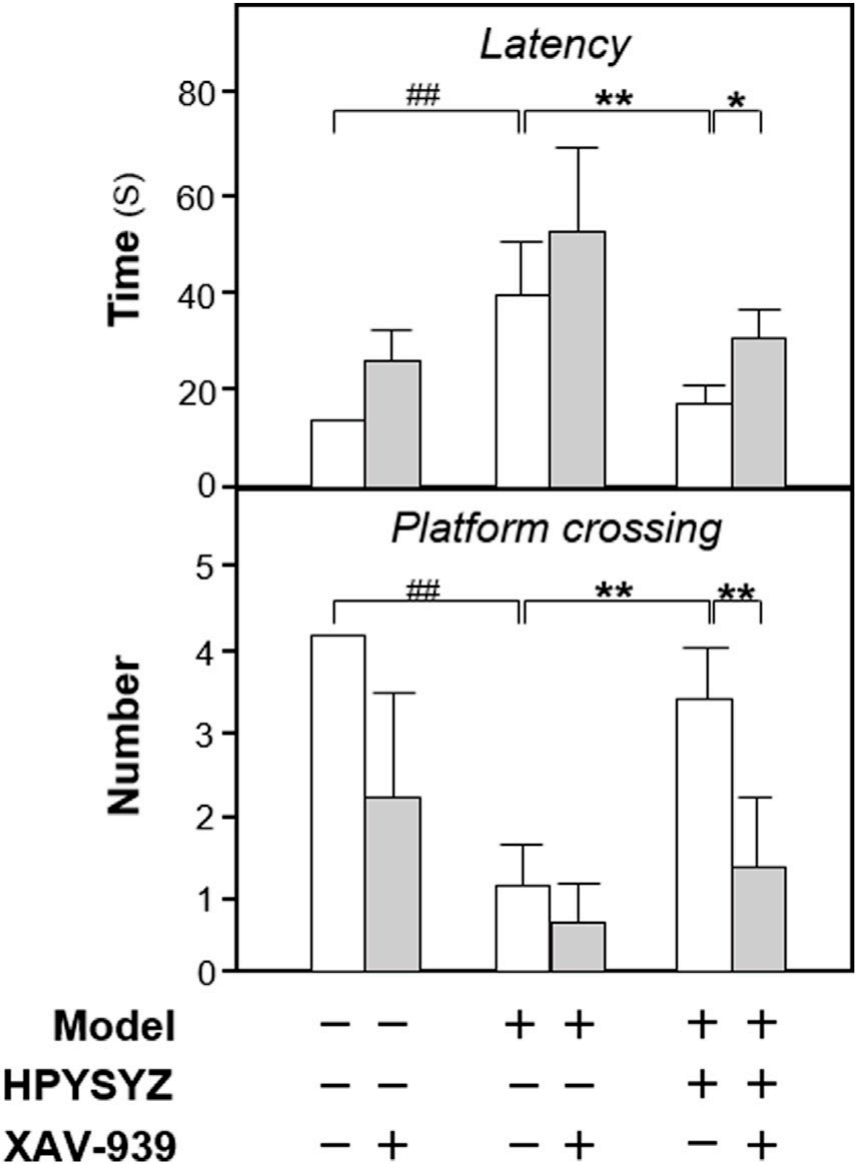
Effect of co-treatment of XAV-939 and ethyl acetate extracts of succinum on learning and memory abilities of carotid artery ligation rats. The effect of co-treatment of XAV-939 and ethyl acetate extracts of succinum on learning and memory abilities on carotid artery ligation rats were evaluated by determining the latency time and platform crossing number of water maze test. Values are expressed as mean ± SEM (*n* = 8). Comparisons between groups were carried out by a one-way ANOVA followed by a *post hoc* Bonferroni test. ^##^ *p* < 0.01, ^*^ *p* < 0.05, and ^**^ *p* < 0.01.

To validate the role of the GSK3β/β-catenin signaling pathway of ethyl acetate extracts of succinum in improving learning and memory impairment of carotid artery ligation rats, the inhibitor XAV-939 was applied for the study. As shown in Figure 6, the time of latency in the model group was increased, while the number of platform crossing was decreased compared with the sham group (*p* < 0.01). Compared with the carotid artery ligation model rats, ethyl acetate extracts of succinum decreased the latency time and increased the platform crossing number of the model animals (*p* < 0.01). Compared with the single ethyl acetate extracts of the succinum treatment group, the combined treatment of XAV-939 and ethyl acetate extracts of succinum increased the time of latency and decreased the number of platform crossing, which suggested that the improvement of ethyl acetate extracts of succinum on the learning and memory abilities of carotid artery ligation rats was attenuated by XAV-939.

**FIGURE 7:**
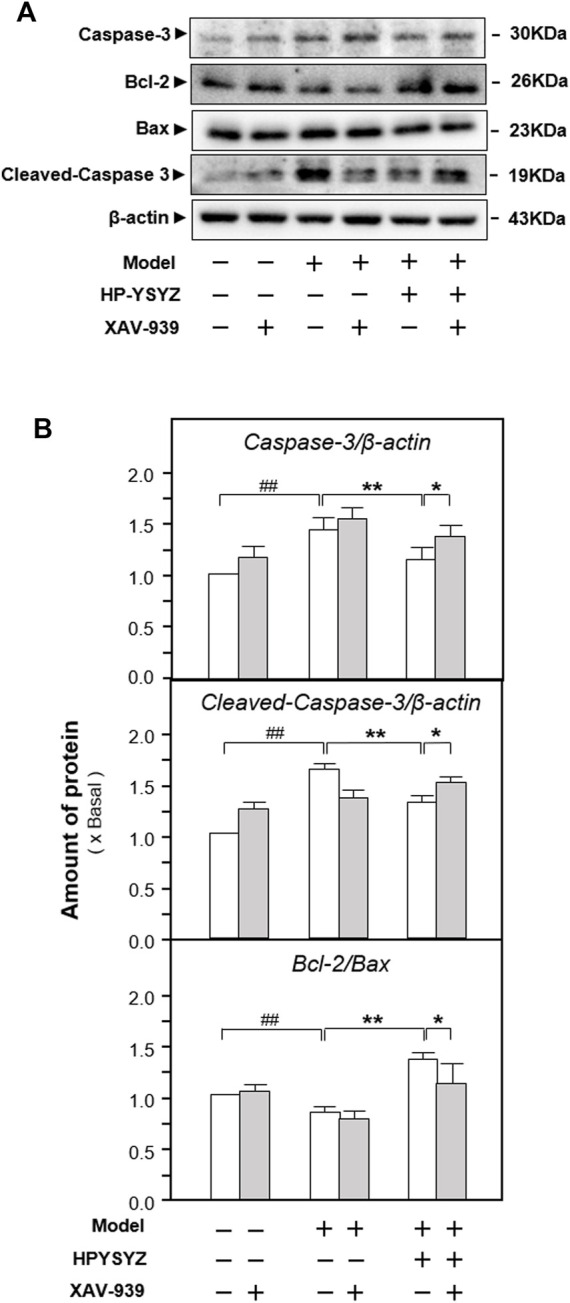
Effect of co-treatment of XAV-939 and ethyl acetate extracts of succinum on the caspase-dependent pathway of hippocampus of carotid artery ligation rats. **(A)** Effect of ethyl acetate extracts of succinum (HPYSYZ) on the expressions of caspase-3, cleaved-caspase-3, Bcl-2, Bax, and β-actin in hippocampus of carotid artery ligation rats after co-treatment of XAV-939 were evaluated by Western blotting analysis. The representative images are displayed (*n* = 5). **(B)** Expression ratio of caspase-3 to β-actin, cleaved-caspase-3 to β-actin and Bcl-2 to Bax were calculated and the expression of β-actin was applied for normalization. Values are expressed as mean ± SEM (*n* = 5). Comparisons between groups were carried out by a one-way ANOVA followed by a *post hoc* Bonferroni test. ^##^ *p* < 0.01, ^*^ *p* < 0.05, and ^**^ *p* < 0.01.

**FIGURE 8:**
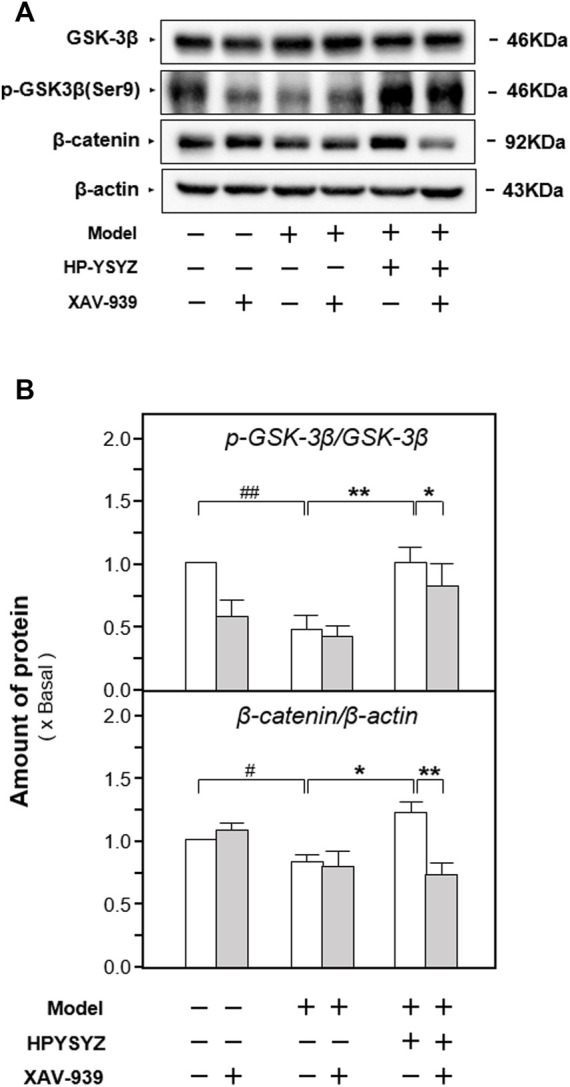
Effect of co-treatment of XAV-939 and ethyl acetate extracts of succinum on the GSK3β/β-catenin pathway of hippocampus of carotid artery ligation rats. **(A)** Effect of ethyl acetate extracts of succinum (HPYSYZ) on the expressions of GSK3β, p-GSK3β (Ser9), β-catenin, p-β-catenin, and β-actin in hippocampus of carotid artery ligation rats after co-treatment of XAV-939 were evaluated by Western blotting analysis. The representative images are displayed (*n* = 5). **(B)** Expression ratio of p-GSK3β to GSK3β was calculated, and the expression of β-actin was applied for normalization. Expression ratio of p-β-catenin to β-catenin was calculated, and the expression of β-actin was applied for normalization. Values are expressed as mean ± SEM (*n* = 5). Comparisons between groups were carried out by a one-way ANOVA followed by a *post hoc* Bonferroni test. ^##^ *p* < 0.01, ^*^ *p* < 0.05, and ^**^ *p* < 0.01.

Moreover, the expressions of proteins related to the caspase-dependent apoptosis pathway and the GSK3β/β-catenin signaling pathway were also determined in the hippocampus of carotid artery ligation rats co-treated with XAV-939 and ethyl acetate extracts of succinum. As shown in Figures 7A,B, single treatment of ethyl acetate extracts of succinum decreased the expression of apoptotic proteins rat compared with the model group (*p* < 0.01). However, the co-treatment of XAV-939 and ethyl acetate extracts of succinum increased the expressions of apoptotic proteins compared with the single ethyl acetate extracts of the succinum treatment group. The similar phenomenon could also be found in determination of the GSK3β/β-catenin signaling pathway-related protein expressions. As shown in Figures 8A,B, single ethyl acetate extracts of succinum increased the expression ratio of p-GSK3β/GSK3β and β-catenin expression in the hippocampus of carotid artery ligation rats (*p* < 0.05). Compared with the single treatment of ethyl acetate extracts of succinum-treated group, the co-treatment of XAV-939 and ethyl acetate extracts of succinum also reversed these action trends. Therefore, these data indicated that ethyl acetate extracts of succinum improves learning and memory abilities on carotid artery ligation rats and reduced the apoptosis of the hippocampus by regulating the GSK3β/β-catenin pathway.

### Effect of Ethyl Acetate Extracts of Succinum on Apoptosis of Neuronal Cells on *In Vitro* Models

**FIGURE 9:**
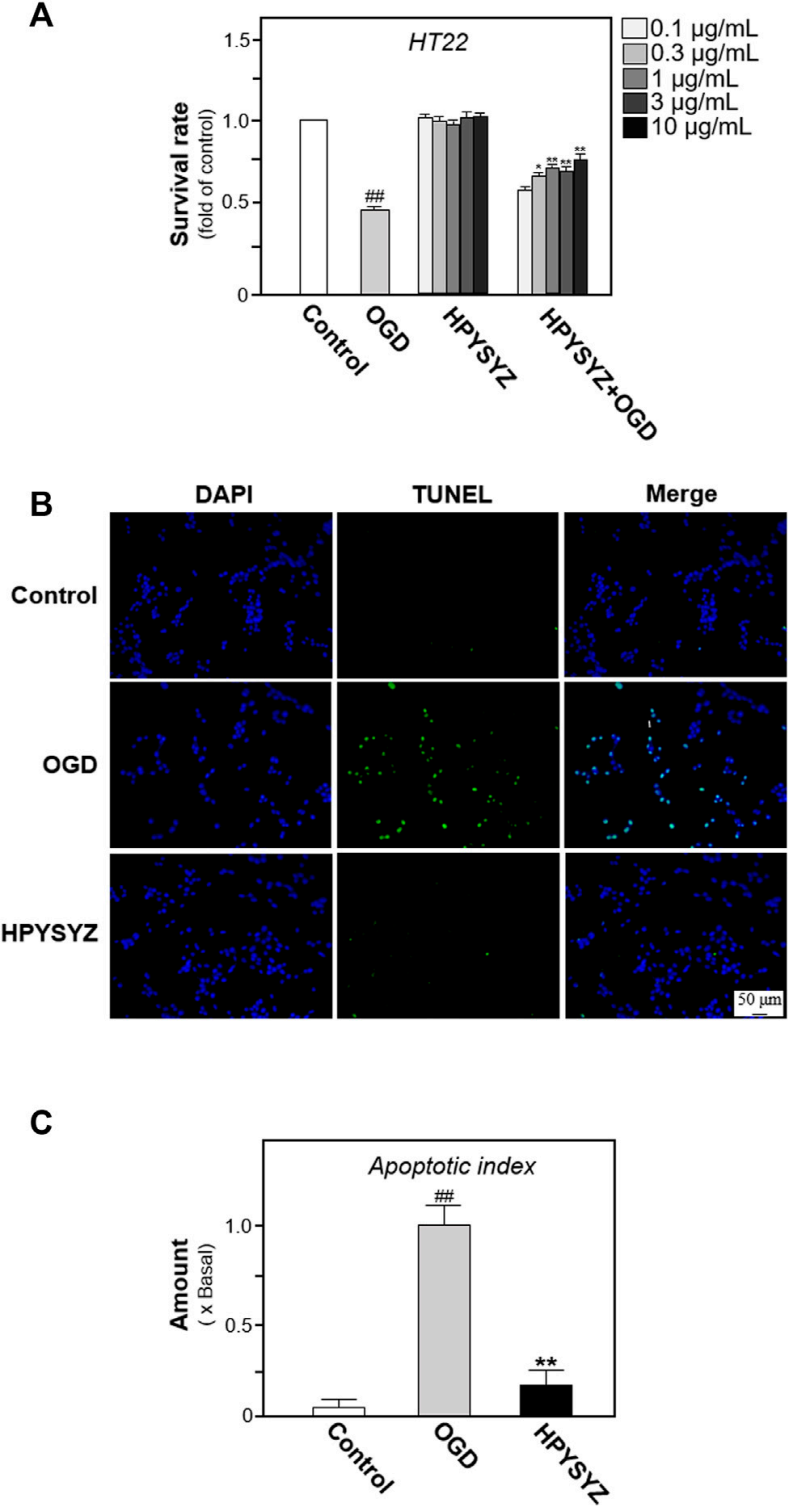
Ethyl acetate extracts of succinum decreased the apoptosis of HT22 cells under OGD condition. **(A)** HT22 cells were treated with ethyl acetate extracts of succinum (HPYSYZ) from 0.1 μg/ml to 10.0 μg/ml for 48 h under OGD condition, and cell viability was determined by MTT assay. **(B)** Treatment of HT22 cells was same as that in **(B)** and the TUNEL assay was applied for evaluation of cell apoptosis. The representative images of the control group, OGD model group, and HPYSYZ treatment (10 μg/ml) are displayed. **(C)** Percentage of TUNEL-staining positive cells to DAPI-staining positive cells was calculated for apoptosis evaluation. Values are expressed as the percentage of the control group as mean ± SEM (*n* = 8). Comparisons between groups were carried out by a one-way ANOVA followed by a *post hoc* Bonferroni test. #*p* < 0.05 (compared with control group); *p < 0.05, and **p < 0.01 (compared with the OGD model group).

**FIGURE 10:**
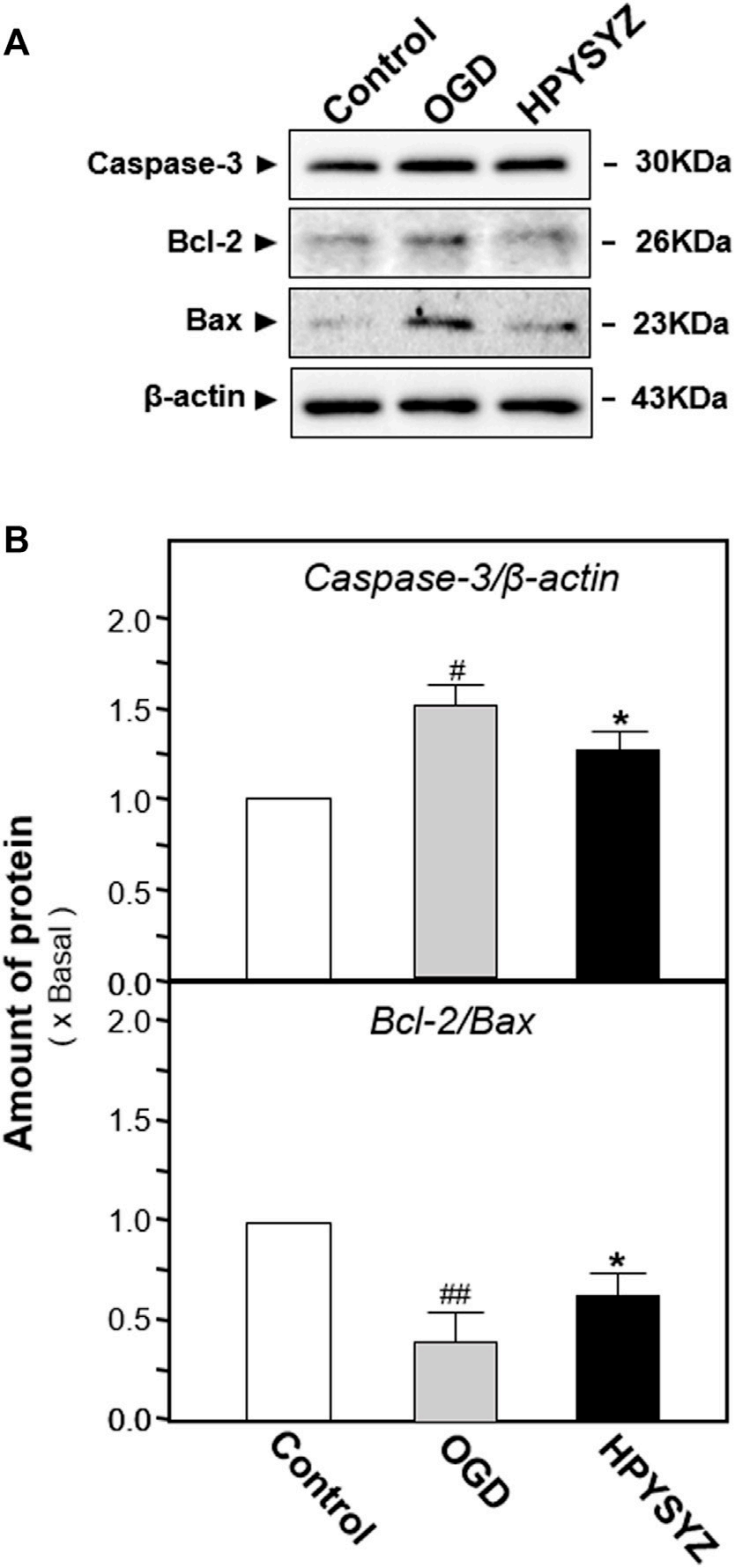
Ethyl acetate extracts of succinum regulated the caspase-dependent pathway of HT22 cells under OGD condition. **(A)** Effects of ethyl acetate extracts of succinum (HPYSYZ) on the expressions of caspase-3, Bcl-2, and Bax of HT22 cells under OGD condition were evaluated by Western blotting analysis. The representative images are displayed (*n* = 5). **(B)** Expression of caspase-3 was analyzed, and the expression of β-actin was applied for normalization. Expressions of Bax and Bcl-2 were analyzed, and the expression of β-actin was also used for normalization. The expression ratio of Bcl-2 to Bax was calculated. Values are expressed as the percentage of the control group (no drug treatment group) as mean ± SEM (*n* = 5). Comparisons between groups were analyzed by a one-way ANOVA followed by a *post hoc* Bonferroni test. ^##^ *p* < 0.01 (compared with control group), *p < 0.05, and **p < 0.05 (compared with the OGD model group).

To further validate this phenomenon and elucidate the possible action mechanism, the oxygen and glucose deprivation (OGD)-treated mouse hippocampal neuronal cell line (also named HT22 cell) mimicking cerebral ischemia inducing neuronal damage was applied for the study. As shown in Figures 9A–C, the cell viability was decreased and the number of apoptotic cells in the OGD model group was increased compared with the control group. Treatment of the ethyl acetate extracts of succinum significantly reversed this trend, which indicated that succinum could ameliorate the apoptosis of HT22 cells under OGD condition. In addition, the effect of ethyl acetate extracts of succinum was also evaluated by determining the expression of apoptosis-related proteins. As shown in Figures 10A,B, the expression of caspase-3 was significantly upregulated, while the ratio of Bcl-2 to Bax was significantly downregulated in the OGD model group compared with the control group (*p* < 0.05), which suggests that OGD condition accelerated HT22 cell apoptosis. We also found that ethyl acetate extracts of succinum significantly reversed this trend, which was manifested in downregulating the expression of caspase-3 and caspase 9 while upregulating the expression raito of Bcl-2 to Bax compared with untreated OGD model group (*p* < 0.05).

### Ethyl Acetate Extracts of Succinum Ameliorated Apoptosis of HT22 Cells Under OGD Condition by Regulating the GSK3β/β-Catenin Pathway

**FIGURE 11:**
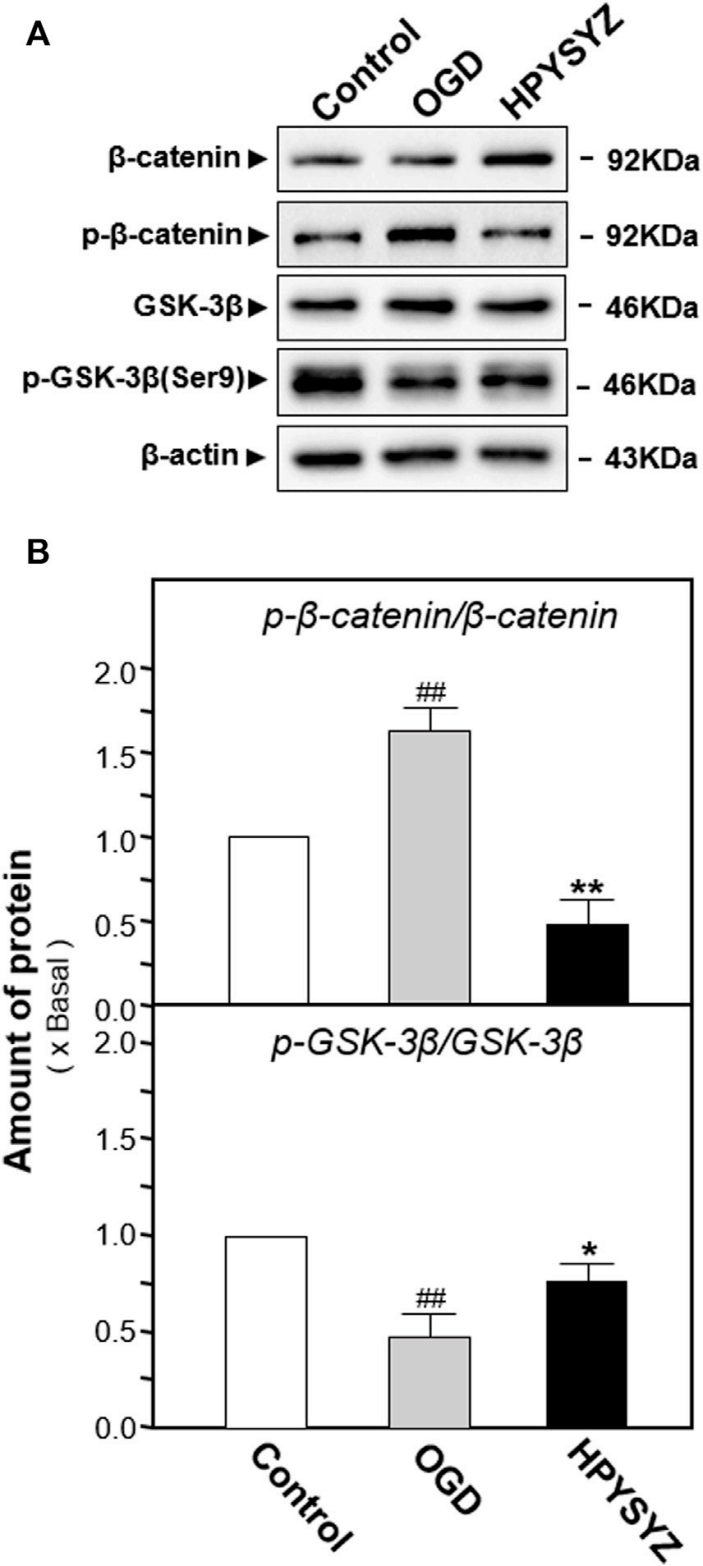
Ethyl acetate extracts of succinum regulated the GSK3β/β-catenin signaling pathway of HT22 cells under OGD condition. **(A)** Effect of ethyl acetate extracts of succinum (HPYSYZ) on the expressions of GSK3β, p-GSK3β (Ser9), β-catenin, p-β-catenin, and β-actin of HT22 cells under OGD condition were evaluated by Western blotting analysis. The representative images are displayed (*n* = 5). **(B)** Expression ratio of p-β-catenin to β-catenin was calculated, and the expression of β-actin was applied for normalization. Expression ratio of p-GSK3β (Ser9) to GSK3β was calculated, and the expression of β-actin was applied for normalization. Values are expressed as the percentage of the control group (no drug treatment group) as mean ± SEM (*n* = 5). Comparisons between groups were analyzed by a one-way ANOVA followed by a *post hoc* Bonferroni test. ^##^ *p* < 0.01 (compared with the control group), *p < 0.05, and **p < 0.05 (compared with the OGD model group).

The effects of ethyl acetate extracts of succinum on regulating the GSK3β/β-catenin signaling pathway on the OGD-treated HT22 cells were also evaluated by determining the related kinase expressions. As shown in Figures 11A,B, the ratio of p-GSK3β (ser9) to GSK3β in the OGD model cells was decreased while the ratio of p-β-catenin to β-catenin was increased compared with the untreated control group (*p* < 0.01). This action trend was successfully reversed by the treatment of ethyl acetate extracts of succinum.

**FIGURE 12:**
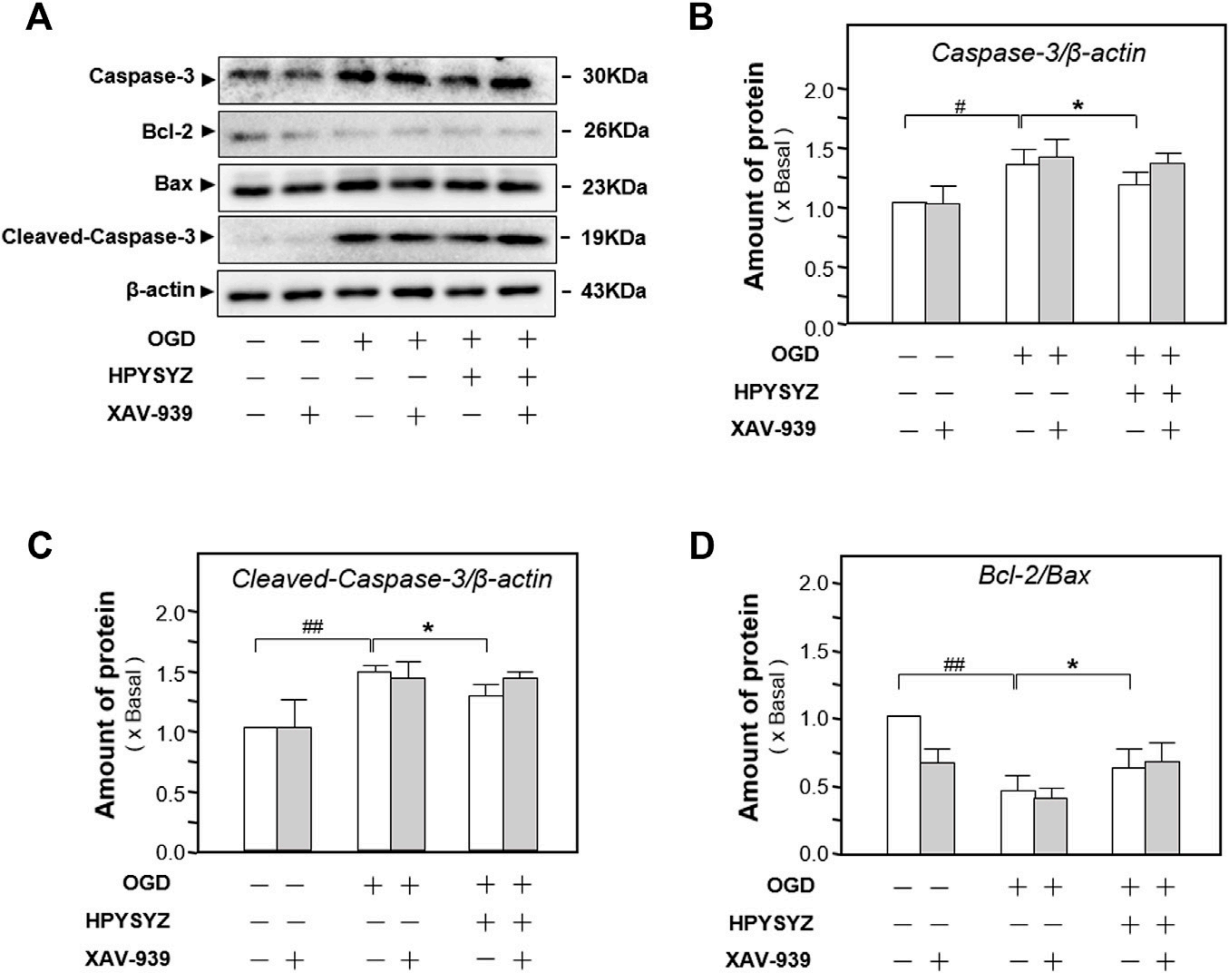
Effect of co-treatment of ethyl acetate extracts of succinum and XAV-939 on regulating the caspase-dependent pathway of HT22 cells under OGD condition. **(A)** Effect of ethyl acetate extracts of succinum (HPYSYZ) on the expressions of caspase-3, Bcl-2, Bax, and β-actin of HT22 cells under OGD condition after the co-treatment of XAV-939 was evaluated by Western blotting analysis. The representative images are displayed (*n* = 5). **(B)** Expression ratio of caspase-3 to β-actin was calculated, and the expression of β-actin was applied for normalization. **(C)** The expression ratio of Cleaved-Caspase 3 to β-actin was calculated and the expression of β-actin was applied for normalization. **(D)** Expression ratio of Bcl-2 to Bax was calculated, and the expression of β-actin was applied for normalization. Values are expressed as mean ± SEM (*n* = 5). Comparisons between groups were carried out by a one-way ANOVA followed by a *post hoc* Bonferroni test. ^##^ *p* < 0.01, ^*^ *p* < 0.05, and ^**^ *p* < 0.01.

**FIGURE 13:**
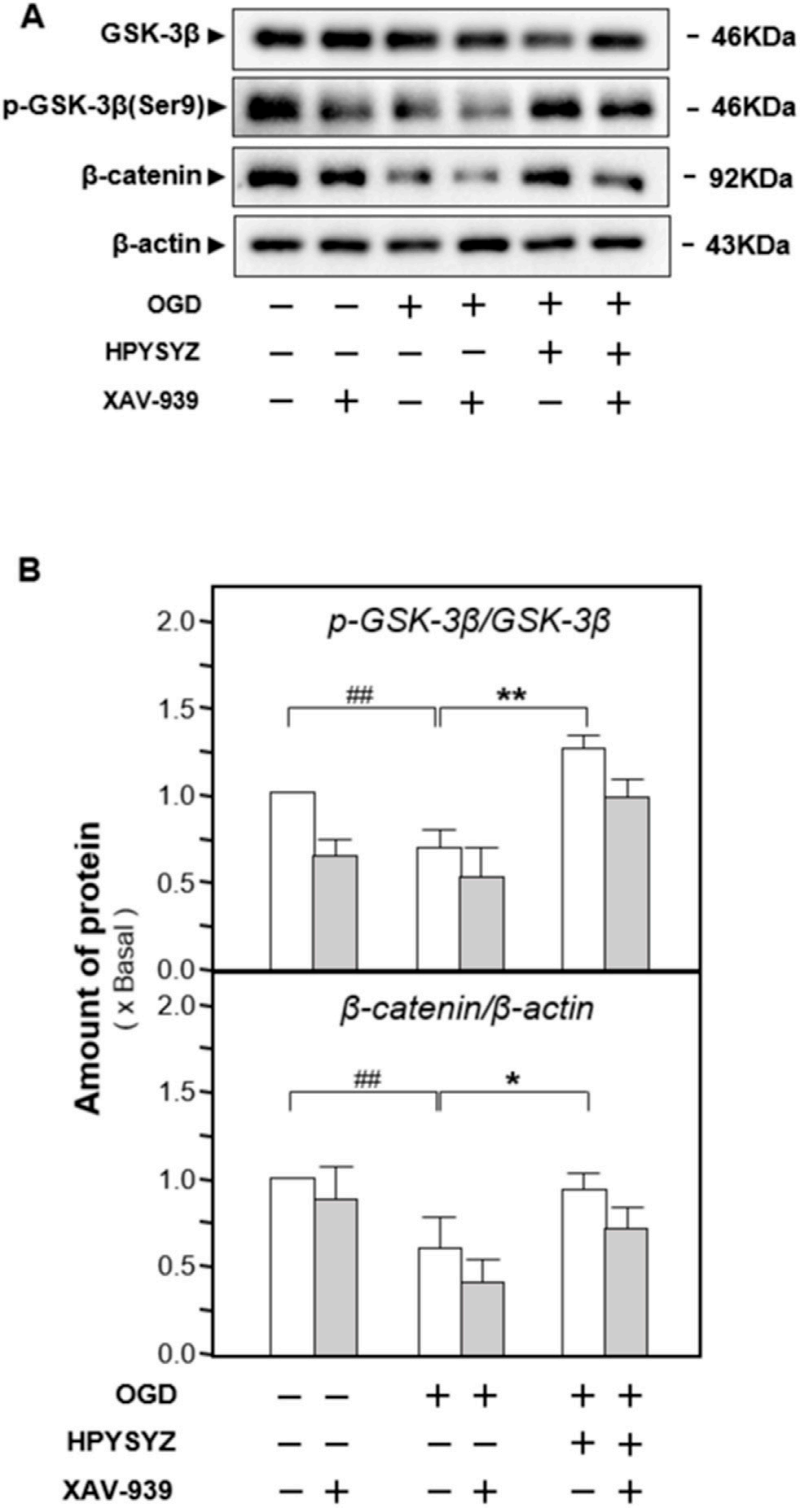
Effect of co-treatment of XAV-939 and ethyl acetate extracts of succinum on regulating the GSK3β/β-catenin pathway of HT22 cells under OGD condition. **(A)** Effect of ethyl acetate extracts of succinum (HPYSYZ) on the expressions of GSK3β, p-GSK3β (Ser9), β-catenin, and β-actin of HT22 cells under OGD condition were evaluated by Western blotting analysis. The representative images are displayed (*n* = 5). **(B)** Expression ratio of p-GSK3β to GSK3β was calculated, and the expression of β-actin was applied for normalization. Expression ratio of p-β-catenin to β-catenin was calculated, and the expression of β-actin was applied for normalization. Values are expressed as mean ± SEM (*n* = 5). Comparisons between groups were carried out by a one-way ANOVA followed by a *post hoc* Bonferroni test. ^##^ *p* < 0.01, ^*^ *p* < 0.05, and ^**^ *p* < 0.01.

Furthermore, the inhibitor of the GSK3β/β-catenin pathway XAV-939 was also co-treated with ethyl acetate extracts of succinum on HT22 cells under OGD condition to validate the signaling pathway. As shown in Figures 12A,B, single treatment of ethyl acetate extracts of succinum downregulated the expressions of apoptotic proteins on HT22 cells under oxygen–glucose deprivation (*p* < 0.05). Compared with the single treatment of ethyl acetate extracts of the succinum group, co-treatment of XAV-939 and ethyl acetate extracts of succinum upregulated the expressions of these proteins of the caspase-dependent pathway, which implied that the effect of ethyl acetate extracts of succinum ameliorating the apoptosis of the OGD-treated HT22 cell was attenuated. Moreover, as shown in Figures 13A,B, single treatment of ethyl acetate extracts of succinum increased the expression ratio of p-GSK3β to GSK3β and β-catenin expression on HT22 cells under OGD condition (*p* < 0.05). Compared with the single treatment of ethyl acetate extracts of the succinum group, co-treatment of XAV-939 and ethyl acetate extracts of succinum decreased the expression ratios of p-GSK3β to GSK3β and β-catenin expression. The ethyl acetate extracts of succinum and XAV-939 had an antagonistic effect on the expression of apoptotic proteins. Taken these data together, regulation of the GSK3β/β-catenin pathway plays a pivotal role in ethyl acetate extracts of succinum-ameliorating HT22 cell apoptosis under OGD condition.

## Discussion

In the current study, we established a VD rat model by carotid artery ligation and found that ethyl acetate extracts of succinum significantly improved cognitive impairment and neuronal cell survival in the hippocampus of model rats. In the HT22 cells, ethyl acetate extracts of succinum reduced the expression of apoptotic proteins such as caspase-3 and Bcl-2/Bax under OGD injury and activated the GSK3β/β-catenin signaling pathway, which might be the action mechanism of succinum in the treatment of VD.

It is now recognized that cerebrovascular dysfunction is an important pathological factor of VD, and ischemia and hypoxia caused by cerebrovascular dysfunction cause cognitive decline. The hippocampus is closely associated with learning and memory, which is particularly vulnerable to ischemic damage (Zhu et al., 2018). Ischemia and hypoxia of brain lead to the accumulation of free radicals and excitatory amino acids in the blood supply area of middle cerebral artery such as the cortex, hippocampus, and striatum, which resulted in intracellular calcium overload and neuronal apoptosis even necrosis (van der Flier et al., 2018; Wang et al., 2020; Kuang et al., 2021). Animal studies have also confirmed that chronic ischemia and hypoxia affecting the survival and growth of hippocampal neurons (Lana et al., 2020). In addition, neuronal damage will further activate microglia and induce pro-inflammatory cytokine release, exacerbating neuronal inflammation and reducing neuronal survival (Qu et al., 2021). Meanwhile, the breakdown of blood–brain barrier leads to the penetration of plasma proteins, neurotoxic compounds, and peripheral immune cells into the brain, which aggravates the damage and eventually causes neurological defects (Lecordier et al., 2021). The GSK3β/β-catenin signaling pathway plays an important role in numerous biological processes including neuronal cell growth and proliferation and is also regarded as an important signaling pathway in the pathological mechanism of VD (Foulquier et al., 2018). In detail, Wnt binds to the related receptor and activates the pathway by inhibiting GSK-3 polymers, leading to phosphorylation of GSK-3β and removal of β-catenin inhibition. β-catenin dissociates from the complex and accumulates into the nucleus to regulate transcription of downstream gene related to antiapoptosis (Xu et al., 2019). It has been found that inactivation of Wnt pathway signaling was closely associated with loss of object recognition memory after unilateral common carotid artery occlusion (Kim et al., 2020). The activation of Wnt/β-catenin signaling pathway promotes angiogenesis under ischemic–hypoxic pathology and alleviates cognitive impairment caused by chronic cerebral under perfusion (Yang et al., 2020). In our study, we found that ethyl acetate extracts of succinum upregulated the expression ratio of p-GSK3β to GSK3β and downregulated the ratio of p-β-catenin to β-catenin, which suggest that ethyl acetate extracts of succinum might regulate the Wnt/GSK3β/β-catenin pathway in the hippocampus of carotid artery ligation rats. This effect was further consolidated by the application of XAV-939. XAV-939 is a small molecule inhibitor of the Wnt/β-catenin signaling pathway that inhibits the expression of Wnt target genes, blocks Wnt signaling upstream of β-catenin, and decreases β-catenin transcript levels by stabilizing axial proteins (Huang et al., 2009; Chen et al., 2014). In current study, the effect of ethyl acetate extracts of succinum on improving learning and memory of carotid artery ligation rats was weakened by XAV-939 treatment, which consolidated our hypothesis.

Most of current drugs treated for VD are Alzheimer’s disease therapeutics such as acetylcholinesterase inhibitors like donepezil, galantamine, and huperzine A, glutamate NMDA receptor antagonists like memantine and calcium antagonists like nimodipine and butalbital (Farooq et al., 2017; Wang, 2020; Song et al., 2021). Memantine ameliorates spatial memory deficits in C57BL/6 mice exposed to chronic ethanol by reducing hippocampal apoptosis (Wang et al., 2018). Donepezil reduces injury after ischemic stroke by stimulating neurogenesis, inhibiting inflammation and apoptosis (Madani Neishaboori et al., 2021). Huperzine A is also popularly applied for the treatment of VD, especially in China. It is proven that huperzine A can improve cognitive deficits in different forms of dementia and has an effect of improving intelligence in animal studies (Damar et al., 2016). In addition to improve the function of the central cholinergic system, huperzine A also increases the level of dopamine in the brain and exerts multiple neuroprotective effects, which effectively counteracts oxidative stress, apoptosis, mitochondrial function damage, and inflammatory response induced by ischemia and hypoxia. Therefore, huperzine A is used as the positive drug in our animal studies. Although these therapeutic drugs have been accepted as one of the common treatments for VD patients, it cannot be ignored that no special drug for treating VD has been approved and the acetylcholinesterase inhibitor exert many side effects. For example, acetylcholinesterase inhibitor may trigger cholinergic effects with adverse effects such as abdominal pain, gastrointestinal discomfort, dry mouth, blurred vision, and weakness. More seriously, acetylcholinesterase inhibitors are strictly prohibited in patients with cardiac insufficiency, asthma, and intestinal obstruction due to the cholinoid effect (Zeng et al., 2015).

Chinese medicine has accumulated rich experiences in the treatment of VD and also has good efficacy. For example, the Shenzhi Jiannao formula and natural medicines such as *Acorus tatarinowii*, *Ligusticum wallichii*, *Salvia miltiorrhiza*, and *Panax ginseng* have been proven to be used in the treatment of VD (Bai and Zhang, 2021). Succinum is also a herbal medicine frequently used for treating disease with “phlegm-obsessed mind, speech like a fool and forgetfulness” in the Qing Dynasty internal medicine book. In particular, its efficacy characteristics of “calming the mind” and “activating blood circulation and dispersing blood stasis” also more conform to the TCM treatment of vascular dementia. However, the modern research on succinum is mainly on focused on epilepsy, while the studies on VD are limited. According to the literature, kujiol A and kujigamberol B isolated from Japanese Kuji Succinum are Ca^2+^ signal transduction inhibitors (Abe et al., 2016; Uchida et al., 2018). It is well known that Ca^2+^ plays an important role in the pathophysiological process of neurodegenerative diseases as a second messenger. Studies have also demonstrated that ligands of the Wnt pathway promote neurodevelopment and differentiation by regulating Ca^2+^-dependent pathways (Shu et al., 2018). Our group previously found that ethyl acetate extracts of succinum significantly prolonged the latency, shortened the duration, and reduced the epilepsy grade in mice caused by pentylenetetrazol, which suggested that ethyl acetate extracts of succinum is the most effective form of succinum (Zhu Z. et al., 2019). In the model of vascular dementia used in our present experiments, ethyl acetate extracts of succinum also showed potency in improving impairment of learning and memory of VD model rats. These findings suggested that ethyl acetate extracts of succinum might be an important fraction with the function of blood activating and blood stasis dispersing. The identification of potent substances of this faction reveals that the isopimarane diterpenes might be the active components. Our study revealed the mechanism of ethyl acetate extracts of succinum extracts in improving cognitive impairment of carotid artery ligation rats. However, the details of the regulation of the GSK3β/β-catenin pathway need to be further clarified. Moreover, the compounds of the ethyl acetate extracts of succinum extracts in improving the learning and memory abilities and modulating the pathway of carotid artery ligation rat will be further elucidated. According to these studies, we hope to provide more scientific basis for the treatment of VD by succinum.

## Conclusion

Ethyl acetate extracts of succinum extracts exerted an anti-VD effect by improving the learning and memory abilities in carotid artery ligation. This effect may be related to the inhibition of neuronal apoptosis in the hippocampus and HT22 cells under OGD condition by regulating the GSK-3β/β-catenin signaling pathway. This study will be helpful for the antivascular dementia alternative therapies or drug developments.

## Data Availability

The original contributions presented in the study are included in the article/Supplementary Material; further inquiries can be directed to the corresponding authors.
