# Supplementary figures and images for: Chinese Medicine, Succinum, Ameliorates Cognitive Impairment of Carotid Artery Ligation Rats and Inhibits Apoptosis of HT22 Hippocampal Cells via Regulation of the GSK3β/β-Catenin Pathway
Source: Front Pharmacol. 2022 Jun 15;13:867477. doi: 10.3389/fphar.2022.867477 (PMC9240707; doi:10.3389/fphar.2022.867477)

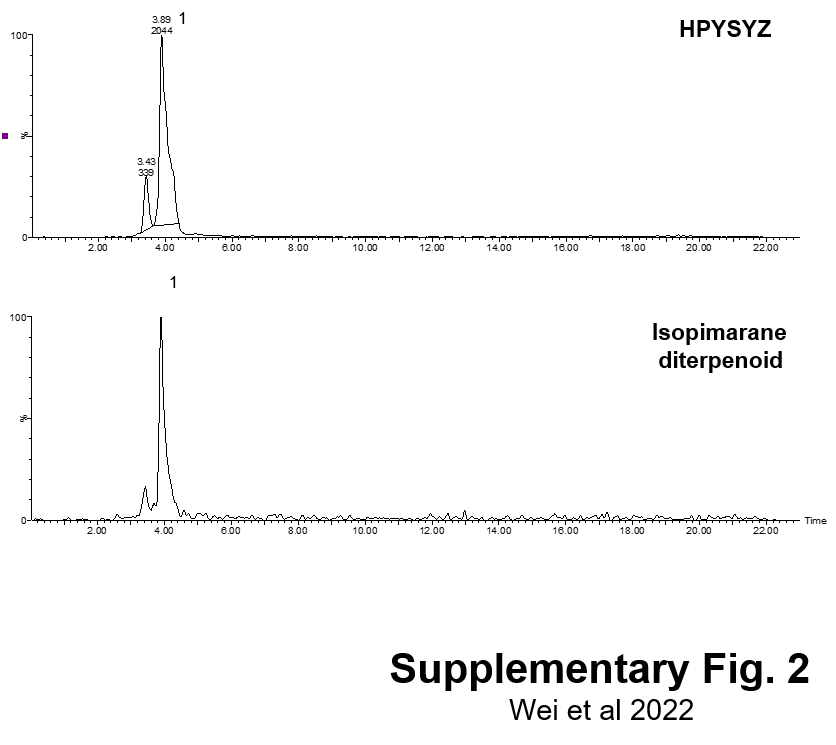

Supplement: Supplementary file 1 [file Image2.TIF]

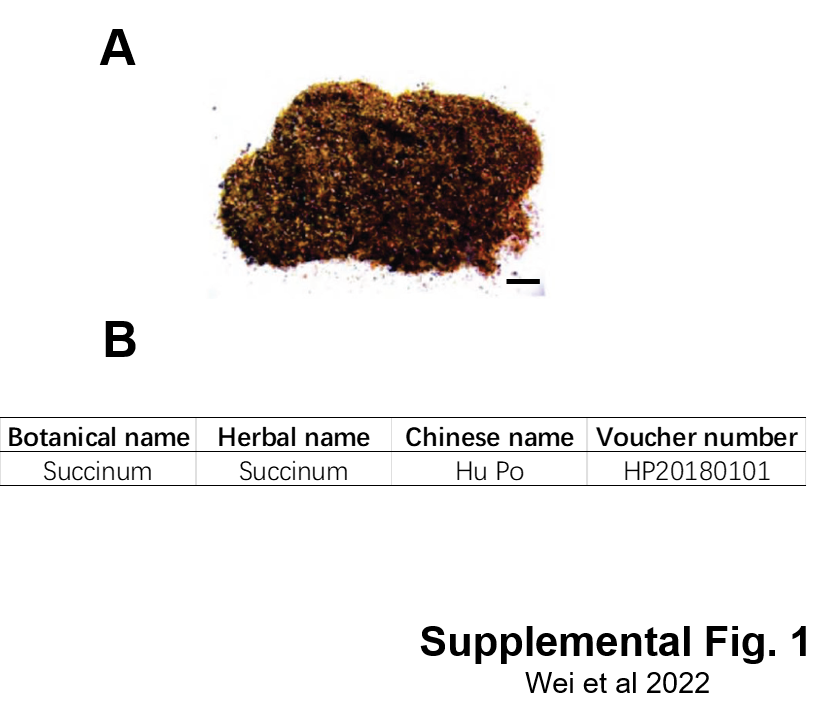

Supplement: Supplementary file 2 [file Image1.TIF]
